# Utilizing Targeted Gene Therapy with Nanoparticles Binding Alpha v Beta 3 for Imaging and Treating Choroidal Neovascularization

**DOI:** 10.1371/journal.pone.0018864

**Published:** 2011-04-29

**Authors:** Hani Salehi-Had, Mi In Roh, Andrea Giani, Toshio Hisatomi, Shintaro Nakao, Ivana K. Kim, Evangelos S. Gragoudas, Demetrios Vavvas, Samira Guccione, Joan W. Miller

**Affiliations:** 1 Angiogenesis Laboratory, Massachusetts Eye and Ear Infirmary, Department of Ophthalmology, Harvard Medical School, Boston, Massachusetts, United States of America; 2 Radiological Sciences Laboratory, Lucas Center, Stanford University, Palo Alto, California, United States of America; Massachusetts Institute of Technology, United States of America

## Abstract

**Purpose:**

The integrin αvβ3 is differentially expressed on neovascular endothelial cells. We investigated whether a novel intravenously injectable αvβ3 integrin-ligand coupled nanoparticle (NP) can target choroidal neovascular membranes (CNV) for imaging and targeted gene therapy.

**Methods:**

CNV lesions were induced in rats using laser photocoagulation. The utility of NP for *in vivo* imaging and gene delivery was evaluated by coupling the NP with a green fluorescing protein plasmid (NP-GFPg). Rhodamine labeling (Rd-NP) was used to localize NP in choroidal flatmounts. Rd-NP-GFPg particles were injected intravenously on weeks 1, 2, or 3. In the treatment arm, rats received NP containing a dominant negative Raf mutant gene (NP-ATPμ-Raf) on days 1, 3, and 5. The change in CNV size and leakage, and TUNEL positive cells were quantified.

**Results:**

GFP plasmid expression was seen *in vivo* up to 3 days after injection of Rd-NP-GFPg. Choroidal flatmounts confirmed the localization of the NP and the expression of GFP plasmid in the CNV. Treating the CNV with NP-ATPμ-Raf decreased the CNV size by 42% (P<0.001). OCT analysis revealed that the reduction of CNV size started on day 5 and reached statistical significance by day 7. Fluorescein angiography grading showed significantly less leakage in the treated CNV (P<0.001). There were significantly more apoptotic (TUNEL-positive) nuclei in the treated CNV.

**Conclusion:**

Systemic administration of αvβ3 targeted NP can be used to label the abnormal blood vessels of CNV for imaging. Targeted gene delivery with NP-ATPμ-Raf leads to a reduction in size and leakage of the CNV by induction of apoptosis in the CNV.

## Introduction

Age-related macular degeneration (AMD) is the leading cause of blindness in developed countries for people over the age of 50 [1]–[3]. The neovascular or “wet” form of the disease, characterized by the development of choroidal neovascular membranes (CNV) is the main cause of visual impairment in macular degeneration [3]–[5]. With the advent of new treatment options such as photodynamic therapy, and especially intravitreal antiangiogenic pharmacotherapy, the visual prognosis of patients with CNV has improved significantly [6]–[9]. However, the current standard-of-care therapies require monthly intravitreal injections by a retina specialist due to their short half-life in the vitreous [10], [11]. Aside from the logistic difficulties and the patients' discomfort, it also puts the patient at risk for cataract formation, endophthalmitis, vitreous hemorrhage, and retinal detachment. Thus, there is a great need for alternative means of delivering antineovascular therapy to the retina.

Recently, there has been substantial progress in the development of nanoparticles with an integrin-targeted delivery system [12]–[15]. During vascular remodeling and angiogenesis, several integrins are expressed on the endothelial cells to potentiate cell invasion and proliferation [16], [17]. Among them, integrin αvβ3 is expressed on many cell types but its expression level in normal tissue is generally low [18], [19]. It is preferentially expressed on angiogenic blood vessels, mediating survival signal and facilitating vascular cell proliferation [20], [21]. Previous reports show that integrin αvβ3 is involved in ocular angiogenesis [22], [23]. *In vivo* experiments have shown antibodies blocking or immunoconjugate drug therapy targeting integrin αvβ3 inhibit neovascularizaion [17], [23]–[26]. In addition, integrin αvβ3 potentiates the internalization of various viruses [27], [28], making it a potential target for drug delivery via liposome based nanoparticles.

Previously we have shown that systemic injection of a cationic nanoparticle coupled to an integrin αvβ3-targeting ligand (NP) can deliver a suicide gene to the tumor neovasculature in rats, causing apoptosis and significant tumor regression [12]. Here we evaluated and were able to demonstrate that NP can target choroidal neovascular membranes (CNV) in rats for imaging and targeted gene therapy using a plasmid DNA encoding ATPμ-Raf, a dominant-negative mutant form of Raf kinase [29].

## Materials and Methods

### Animals and Ethics Statement

All experiments were conducted in accordance with the recommendations in the Guide for the Care and Use of Laboratory Animals of the National Institutes of Health.and the guidelines established by the Animal Care Committee (ACC) of the Massachusetts Eye and Ear Infirmary. The protocol was approved by the ACC (protocol number 07-10-012). A total of 106 Brown-Norway male rats weighing 175–225 grams were obtained from Charles River Laboratories (Wilmington, MA) and used for the experiments.

### Characteristics and preparation of Nanoparticles

Detailed description of the NPs and their synthesis has been published previously [12]. All custom-made lipids and genes were GLP manufactured. Briefly, purified lipid components were dissolved in organic solvents (CHCl3 and CH3OH in a ratio 1∶1). The CHCl_3_ and CH_3_OH were evaporated and dried in rotavap for 24 hours. Distilled and deionized water was added to yield a heterogeneous solution of 30 mM in total lipid concentration. The lipid/water mixture was then sonicated with a probe-tip sonicator for at least one hour. Throughout sonication, the pH of the solution was maintained between 7.0 and 7.5 with 0.01N NaOH solution, and the temperature was maintained above the gel-liquid crystal phase transition point (Tm). The liposome solution was transferred to a petri dish resting on a bed of wet ice, cooled to 0°C, and irradiated at 254 nm for at least one hour with a hand-held UV lamp placed 1 cm above the petri dish, yielding NPs. The NPs were then filtered through a 0.2 µm filter and collected.

Using a Brookhaven dynamic light scattering system (DLS), the size (diameter), distribution, and zeta potential of NPs were determined to be 45.3^+^2.4 nm and +35mv respectively, averaged for 17 cycles of NP synthesis.

The rats received intravenous treatments at a dose 1 mg/kg of NP and 1 µg/kg of plasmid DNA containing the Raf mutant gene (ATPμ-Raf). Total volume of injection was 350 µl.

### Induction of Choroidal Neovascular Membranes

Animals were anesthetized with an intraperitoneal injection of 0.2 to 0.3 mL of a 1∶1 mixture of 100 mg/mL ketamine and 20 mg/mL xylazine. Pupils were dilated with 5.0% phenylephrine and 0.8% tropicamide. CNV was induced in the eyes of rats with a 532-nm laser (Oculight GLx; Iridex, Mountain View, CA), as previously described [30]–[32]. Four to eight laser spots (180 mW, 100 µm, 100 ms) were placed in each eye of the rat with a slit lamp delivery system and a cover slip serving as a contact lens. If significant hemorrhage occurred, the eye was excluded.

### Evaluation of Specific Targeting of CNV Using *In Vivo* Imaging and Choroidal Flatmounts

NP carrying a green fluorescing protein (GFP) plasmid (NP-GFPg) was used to evaluate the ability of the particles to deliver a gene to the neovascular endothelial cells. Rhodamine labeling of the NP (Rd-NP) was used to localize the particles in choroidal flatmounts. Rats were divided into 3 groups of 6 animals and evaluated 1, 2, or 3 weeks after creation of CNV. The formation of CNV was confirmed by fluorescein angiography (FA) using a digital fundus camera (TCR50IA; Topcon, Paramus, NJ) following intraperitoneal injection of 0.2 mL of 2% fluorescein sodium. The rats where then injected with Rd-NP-GFPg particles. The expression of GFP was evaluated with *in vivo* imaging using the same camera and FA filter settings 24, 48, and 72 hours after injection of particles. The rats were then euthanized and choroidal flat mounts were performed at the above time points. RPE-choroid-sclera complex was flatmounted (Vector Laboratories, Burlingame, CA) and coverslipped. Pictures of the choroidal flatmounts were taken by a confocal microscope (Leica Microsystems, Wetzler, Germany). Negative controls were evaluated under identical conditions without injection of NP or following injection of non-targeted rhodamine labeled liposome particles carrying GFP-plasmid.

### Treatment of CNV with Targeted Gene Delivery

After creation of laser-induced CNV, animals were divided in to 7 groups; groups A–C were treatment groups and groups D–G were controls. Group A (n = 6 received one intravenous injection of α_ν_β_3_ targeted-NP containing ATPμ-Raf (NP-ATPμ-Raf) on days 1, 3, and 5 after CNV creation; group B (n = 6) received one intravenous injection of NP-ATPμ-Raf on days 3, 5, and 7; group C (n = 3) received one intravenous injection of NP-ATPμ-Raf on days 7, 9, and 11; group D (n = 6) did not receive any treatment; group E (n = 3) received one intravenous injection of non-targeted NP containing ATPμ-Raf (ntNP-ATPμ-Raf) on days 1, 3, and 5; group F (n = 3) received one intravenous injection of α_ν_β_3_ targeted-NP without ATPμ-Raf (NP) on days 1,3, and 5; and group G (n = 3) received one intravenous injection of ATPμ-Raf gene without NP on days 1, 3, and 5.

### Evaluation of CNV Size and Leakage

We used FA and OCT to monitor CNV development and changes *in vivo* and choroidal flatmounts to study the size of the lesions *ex vivo*. FA was performed as detailed above, 1 and 2 weeks after CNV creation in all treated and control animals. A choroidal neovascular membrane was defined as fully regressed after treatment if there was no leakage in the area of treated membrane [30], [31]. The angiograms were graded by two masked readers using a pre-established grading scheme [33], [34]. Briefly, the description of each grade follows: 0, faint hyperfluorescence or mottled fluorescence without leakage; 1, hyperfluorescent lesion without progressive increase in size or intensity; 2A, hyperfluorescence increasing in intensity but not in size; 2B, hyperfluorescence increasing in intensity and in size.

Two weeks after CNV creation, the size of the CNV lesions was measured in choroidal flatmounts using the methods reported previously after perfusion of 5 mg/mL fluorescein labeled dextran [31], [35]. A computer program (OpenLab; Improvision, Boston, MA) was used by two masked investigators to measure the hyperfluorescent areas corresponding to the CNV lesions.

### Optical Coherence Tomography Measurement of CNV Size

Six rats treated with intravenous NP-ATPμ-Raf on days 1,3 and 5 and 6 control rats without treatment were used for evaluation of CNV size *in vivo* using optical coherence tomography (SDOCT, Bioptigen, Durham, NC) on days 3, 5, and 7 after CNV creation. A volume analysis was performed, using 100 horizontal raster, consecutive B-scan lines, each one composed of 1200 A-scans. The volume size was 2.1×2.1 cm. To evaluate the cross-sectional size of each lesion in OCT images, the sections passing through the center of the CNV were chosen. The center of the lesion was defined as the midline passing through the area of RPE-Bruch's membrane rupture. In order to consistently identify this point, we used the en-face fundus reconstruction tool provided with the Bioptigen SD-OCT system. For each time point, the same spot was used to evaluate the size of the CNV. CNV was outlined from the inner border of the retinal pigment epithelial layer to the top of the lesion and the size was measured using Image J software (http://rsbweb.nih.gov/ij/, last access January 7^th^ 2009).

### Histopathology of CNV Lesions

On day 3,5, 7 and 14 after CNV creation, eyes were enucleated and fixed in 4% paraformaldehyde in phosphate-buffered saline (PBS) for 1 hour and cryoprotected. Serial sections of the eyes were cut at 10 µm thickness on a cryostat (CM1850; Leica, Heidelberger, Nussloch, Germany) at −20 C, and prepared for staining. Terminal dUTP Nick-End Labeling (TUNEL) assay was performed according to the manufacturer's protocol (ApoTag Fluorescein in situ Apoptosis Detection Kit; Chemicon, Temecula, CA) as previously reported [31], [36]. CD31 (1∶100, Serotec, Oxford, UK) antibody was used for visualizing endothelial cells and a mouse monoclonal antibody for ED1, the rat homologue of human CD68 (1∶100, Millipore, Billerica, MA) was used for staining macrophages. Sections were then stained with DAPI (1∶1000, Invitrogen Ltd, Carlsbad, CA, USA) for nuclear staining and mounted with Vecta shield mounting media (Vector Laboratories, Burlingame, CA). Photographs of the CNV were taken with upright fluorescent microscope (DM RXA; Leica, Solms, Germany) and the number of TUNEL positive and ED 1 positive cells were counted.

### Statistical Analysis

All values are presented as mean ± SE. Paired groups were compared using the Wilcoxon t-test. For three groups, data were compared by Kruskal-Wallis test and for two group comparisons, Mann-Whitney U test was used (SPSS statistics 17.0, SPSS Inc., Chicago, IL, USA). A P value of less than 0.05 was considered statistically significant.

## Results

### *In Vivo* imaging Reveals GFP-Plasmid Expression by CNV

**Figure 1:**
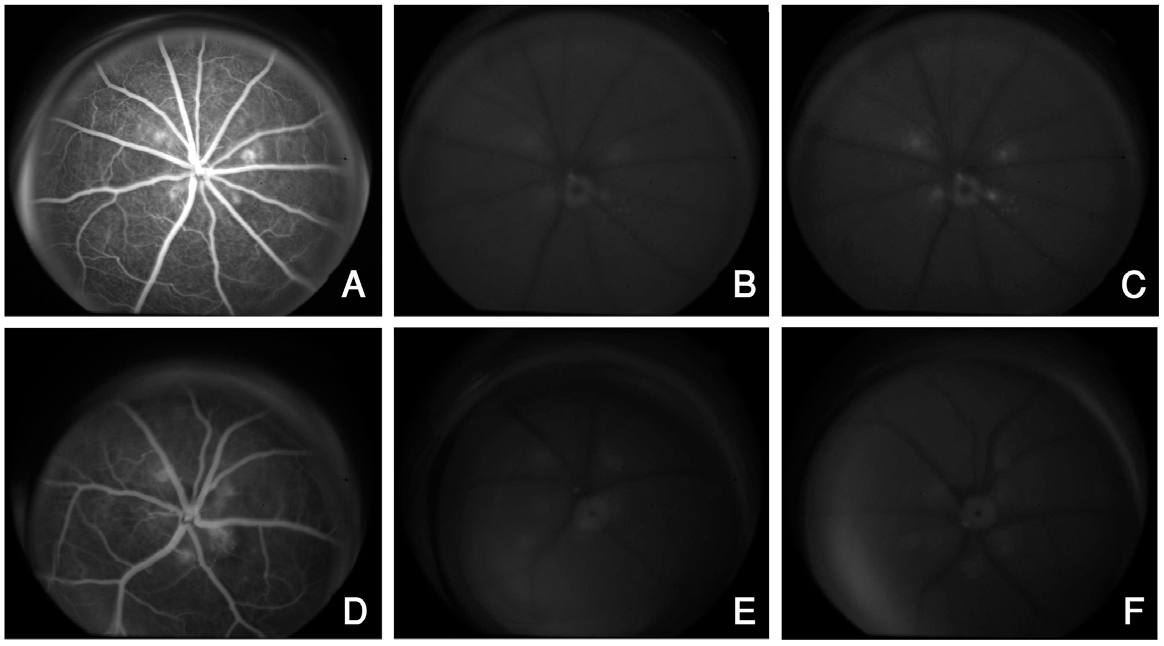
Bioimaging with NP-angiography showing GFP expression using the Topcon camera with fluorescein angiography filter settings. Late phase FAs (A and D) show the CNV lesions prior to injection of NP. Autofluorescent images taken prior to injection of NP reveal minimal background fluorescence of the CNV lesions (B and E). Injection of targeted NP carrying a GFP plasmid (NP-GFPg) causes increased fluorescence of the CNV lesions from GFP expression (C) whereas non-targeted NP carrying a GFP plasmid (ntNP-GFPg) does not cause any increase in the intensity of fluorescence of the CNV over background autofluorescence (F).

Formation of CNV was confirmed by FA prior to injection of NP. One day after injection of rhodamine-labeled αvβ3 targeted nanoparticle carrying a GFP plasmid (Rd-NP-GFPg), digital fundus photography with FA filter settings revealed hyperfluorescence of the CNV lesions, confirming localization and adhesion of the NP and GFP expression. This hyperfluorescence was sustained through day 3 (data not shown) and was seen in 1, 2, and 3-week-old CNV lesions examined (Figure 1A–C). There was no notable hyperfluorescence noted above background fluorescence level in the control groups (Figure 1D–F). There was no evidence of increased fluorescence in the normal retinal or choroidal vasculature.

### Rhodamine-Labeled αvβ3 Targeted Nanoparticle Accumulate in the CNV and Induce GFP Expression

**Figure 2:**
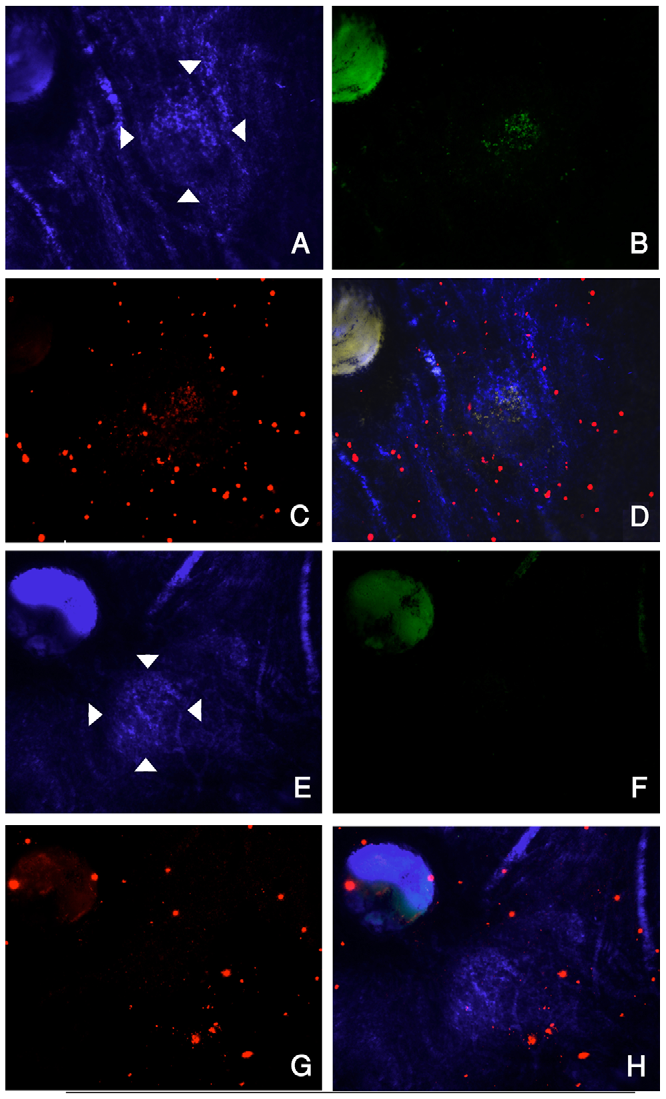
Choroidal flatmounts showing accumulation of rhodamine labeled NP and expression of GFP plasmid in the CNV. The CNV lesions are delineated by arrowheads in bright field images with false blue color (A and E). FITC-filtered images highlight the GFP expression one day after systemic injection of Rd-NP-GFPg (B) whereas non-targeted NP (Rd-ntNP-GFPg) does not induce GFP expression in CNV (F). Cy3-filtered images highlight that rhodamine-labeled NP (Rd-NP-GFPg) accumulates in the CNV (C), while rhodamine-labeled non-targeted NP (Rd-ntNP-GFPg) does not (G). Some particles can be visualized circulating in the choroidal vessels. Overlay of images A–C is presented in panel D and overlay of E–G is shown in H.

The delivery of rhodamine dye and the expression of GFP plasmid in the CNV were confirmed by performing confocal microscopy on choroidal flat mounts (Figure 2). Choroidal flatmounts performed after *in vivo* imaging revealed accumulation of rhodamine labeled nanoparticles in the CNV lesion (Figure 2C). There was an overlap of GFP expression and the rhodamine accumulation in the CNV (Figure 2D). No notable difference was seen in the pattern of NP accumulation or intensity of GFP expression over the time course examined. There was no evidence of increased fluorescence in the normal choroidal vasculature and no evidence of rhodamine accumulation or GFP expression in the control animal injected with non-targeted rhodamine labeled nanoparticles carrying a GFP plasmid (Rd-ntNP-GFPg; Figure 2E–H).

### ανβ3 Targeted-NP Containing ATPμ-Raf Reduces the Size of CNV

**Figure 3:**
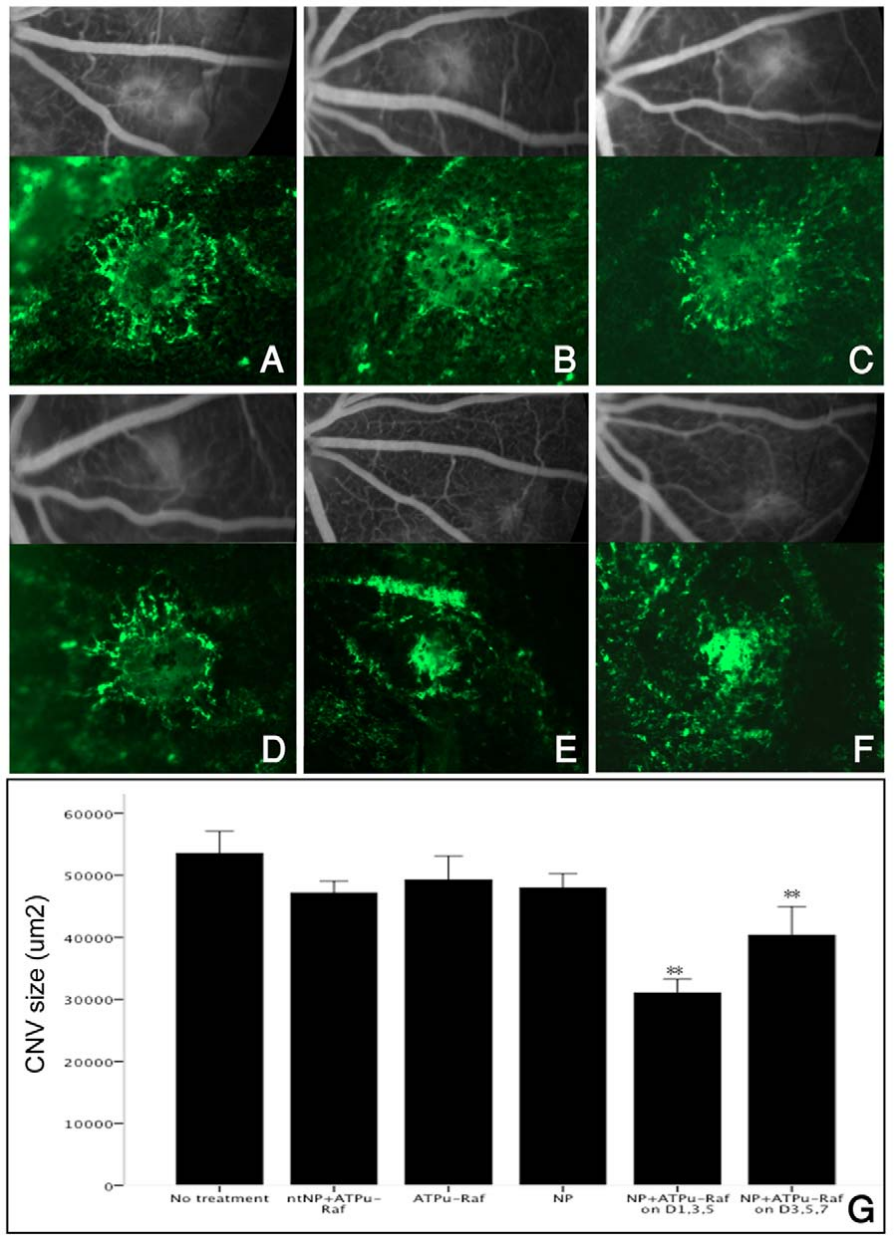
Late phase fluorescein angiography (FA) and choroidal flatmounts (*x10*) two weeks after laser photocoagulation. Representative lesions are from the control group (A–D) and the NP-ATPμ-Raf treated group (E and F). Group (A) received no treatment; (B) received intravenous injection of non-targeted NP containing ATPμ-Raf on days 1, 3, and 5 after laser CNV creation; (C) received intravenous injection of α_ν_β_3_ targeted-NP without ATPμ-Raf gene on days 1,3, and 5; (D) received injection of ATPμ-Raf gene without NP on days 1, 3, and 5; (E) received injection of α_ν_β_3_ targeted-NP containing ATPμ-Raf (NP-ATPμ-Raf) on days 1, 3, and 5; and (F) received injection of NP-ATPμ-Raf on days 3, 5, and 7. NP-ATPμ-Raf treated groups (E and F) had significantly lower grade CNV lesions on FA grading and smaller CNV size compared to the control group (A–D). No statistically significant difference in size was noted between the control groups A–D. Quantification of the CNV size on choroidal flat mounts is shown in (G). *P<0.01. Data are expressed as the mean ± SE.

Treatment of CNV with intravenous injection of ανβ3 targeted-NP containing ATPμ-Raf (NP-ATPμ-Raf) on days 1, 3, and 5 resulted in a 42.0% reduction in the CNV size on choroidal flatmount compared with control CNVs with no treatment (mean size, 53538.7 µm^2^ vs. 31029.3 µm^2^, p<0.001) (Figure 3A and E). Treatment with 3 doses of NP-ATPμ-Raf on days 3, 5 and 7 also showed a 24.6% decrease in the CNV size (Figure 3F). Treatment on days 7, 9, and 11 did not lead to a significant reduction of CNV size (data not shown). Three additional control groups (n = 3 each) were given intravenous injection of non-targeted-NP containing ATPμ-Raf (ntNP- ATPμ-Raf), naked ATPμ-Raf, and ανβ3 targeted-NP with no ATPμ-Raf (NP) on days 1,3, and 5 after CNV formation. There was no difference in the CNV size between any of the 4 control groups, including the no treatment group (Kruskal- Wallis test, p = 0.168) (Figure 3A–D).

**Figure 4:**
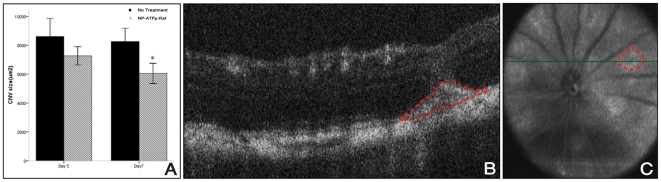
*In vivo* evaluation of CNV utilizing SD-OCT. Quantification of CNV size using SD-OCT (A) reveals a decrease in CNV size, reaching statistical significance on day 7 (Mann- Whitney U test, p = 0.001) in the NP-ATPμ-Raf treated group compared to the control group. A hyper-reflective subretinal lesion is seen as delineated by the red dotted line (B). This lesion corresponds to the hyporeflective area on fundus reconstruction (red dotted circle, C). *P<0.01. Data are expressed as the mean ± SE.

To track the reduction of CNV size *in vivo* after treatment with NP-ATPμ-Raf, we used OCT imaging. We were able to delineate the CNV as a discrete subretinal hyperreflective material starting on day 5 (Figure 4B and C). While the difference in the cross-sectional CNV size on day 5 between treated and control CNV had not reached statistical significance, (Mann- Whitney U test, p = 0.066), significantly decreased size was noted on day 7 in the treated group compared to the control group (Mann- Whitney U test, p = 0.001) (Figure 4A).

### Treatment with NP-ATPμ-Raf Leads to a Reduction in Size and Leakage of CNV on FA

Quantitative assessment of CNV leakage by FA performed on day 14 was carried out by two masked graders. Pathologic leakage from the CNV could be noted in the late phase (6–8 minutes) in the untreated group with large and diffuse area of leakage (Figure 3A–D). The treated group showed less leakage with smaller lesions (Figure 3E and F). FA grading revealed that 70%∼83.8% of the CNV in the different control groups showed grade 2B leakage, while 40.3% of the CNV in the treated group had grade 2B leakage (Table 1; Pearson Chi- square test, p<0.001). There was no difference in the distribution of grading amongst the 4 different control groups (Pearson Chi- square test, p = 0.679). Also, no statistically significant difference was found in the distribution of leakage grading between the groups treated on days 1, 3, and 5 or days 3, 5, and 7 (Pearson Chi- square test, p = 0.152).

**Table 1:** Fluorescein angiography (FA) grading of CNV lesions.

	Grade 1	Grade 2A	Grade 2B	Total
Control	5(6.2%)	16(19.8%)	60(74.1%)	81
ntNP-ATPμ-Raf	2(5%)	10(25%)	28(70%)	40
ATPμ-Raf	3(7.1%)	7(16.7%)	32(76.2%)	42
NP	0(0%)	6(16.2%)	31(83.8%)	37
NP-ATPμ-Raf on D1, 3, 5	10(13.9%)	33(45.8%)	59(40.3%)	72
NP-ATPμ-Raf on D3, 5, 7	1(2.8%)	16(44.4%)	19(52.8%)	36

Significantly higher percentage of control CNV lesions were Grade 2B compared to the treated CNV (Pearson Chi-square test P<0.001). There were no significant differences between the different treatment groups (Pearson Chi-square test P = 0.679).

### Reduced Endothelial Cell Count and Increased Apoptosis in CNV Treated with NP-ATPμ-Raf

**Figure 5:**
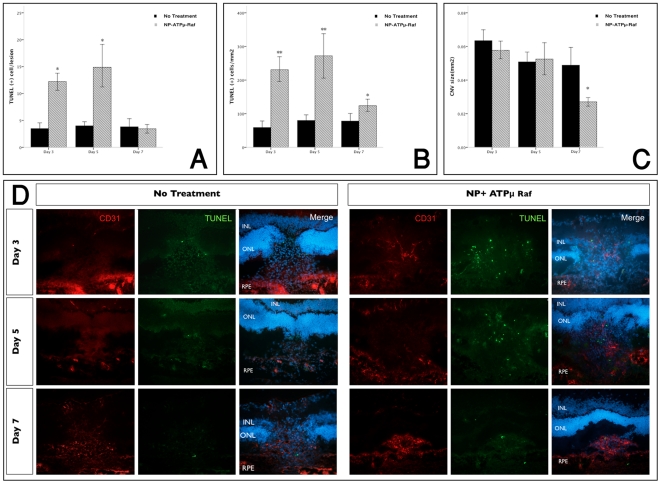
Evaluation of endothelial cell apoptosis with TUNEL staining in frozen sections. Quantification of TUNEL positive cells showed significantly more TUNEL(+) cells/lesion (A) and TUNEL (+) cells/mm^2^ (B) with treatment of NP-ATPμ-Raf compared to the control group on day 3 and 5 after laser injury. There was a statistically significant reduction of CNV size noted on day 7(C). Double-immunofluorescent staining of frozen sections (*x20*) obtained at 3, 5 and 7 days after laser photocoagulation for the endothelial cell marker CD31 and TUNEL stain (D). *P<0.01. Data are expressed as the mean ± SE.

CD31-positive endothelial cells were detected in the subretinal space starting on day 3 after laser injury (figure 5D). The cells increased and focalized to a distinct subretinal membrane representing the CNV by day 7. In the treated CNV, there were fewer CD 31-postive cells and the CNV appeared more compact with distinct borders and fibrous formation by day 7 (Figure 5D).

**Figure 6:**
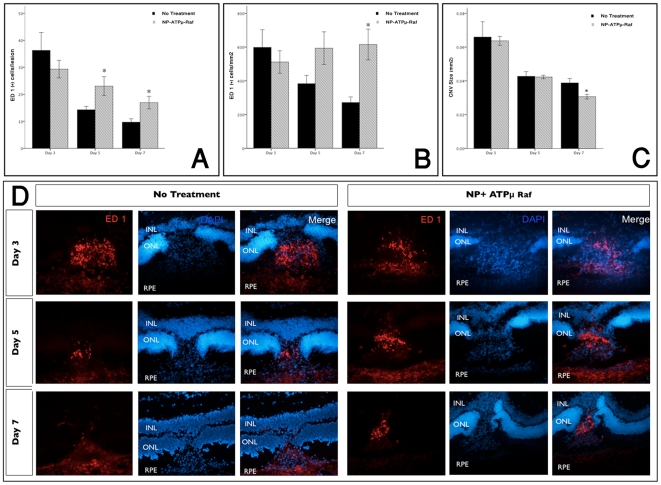
Increased macrophage infiltration at the site of treated CNV. Macrophage infiltration was highest on day 3 with gradual decrease on days 5 and 7. Significantly higher number of macrophages were observed with the NP-ATPμ-Raf treated group compared to the control group on days 5 and 7 (A and B). There was a statistically significant reduction of CNV size noted on day 7(C). Immunofluorescent staining of representative frozen sections (*x20*) obtained at 3, 5, and 7 days after laser photocoagulation for ED 1, a marker for macrophage (D). *P<0.01. Data are expressed as the mean ± SE.

We investigated signs of apoptosis with TUNEL staining. In the treated group, significantly more TUNEL-positive nuclei were observed in the CNV starting on day 3 (Figure 5A and B). This trend continued through day 7 (Mann Whitney U test, p<0.01 for day 3 and 5, p = 0.01 for day 7, Figure 6B). The reduction of the CNV size as measured in histological sections reached statistical significance on day 7 (Mann Whitney U test, P = 0.001, Figure 6C).

### Macrophage infiltration with ανβ3 Targeted-NP Containing ATPμ-Raf

ED 1 positive cells (a marker for macrophages, equivalent to human CD 68) were concentrated within the subretinal space at the laser injury site on day 3 (Figure 6D). No ED 1-positive cells were observed in the undamaged choroid. No difference was noted in the number of ED 1-positive cells infiltrated into the CNV between the treated group and the untreated group on day 3. However, on day 5 and 7 statistically more ED 1-positive cells were seen in the treatment group (Mann Whitney U test, P<0.01 respectively; Figure 6D).

## Discussion

Specific targeting and delivery of medication for the treatment of CNV remains challenging [37]. In this study we were able to target experimental CNV after systemic injection of a cationic nanoparticle coupled to an integrin αvβ3-targeting ligand (NP) and utilize this method for imaging and treatment of CNV in rats.

We first demonstrated the vascular targeting of NP and it's ability to deliver a gene to the neovascular endothelial cells of CNV in rats using rhodamine labeled NPs coupled with GFP (Rd-NP-GFPg). GFP expression was seen *in vivo* using fundus NP-angiography with FA filter settings and *ex vivo* in choroidal flat mounts. *In vivo* imaging revealed increased fluorescence, sustained for 3 days after NP injection, in 1, 2, or 3 week old CNV (figure 1). GFP expression co-localized to the area of rhodamine labeled NP accumulation in the CNV on choroidal flat mounts indicating GFP expression is correlated with areas of NP adhesion to neovascular endothelial cells of CNV (figure 2). None of the retinal or choroidal vasculature showed increased fluorescence from GFP expression. The specific targeting of NP is due to the selectivity of its binding ligand for integrin αvβ3 [12], which has limited cellular distribution in normal tissue including the eye [23], [38]. This integrin is significantly up regulated during the process of vascular remodeling and angiogenesis and is present in pathologic specimens from human eyes with CNV or proliferative diabetic retinopathy (PDR) [17], [22], [23], [39], [40]. With the specific labeling of CNV demonstrated here, fundus NP-angiography utilizing various dyes has the potential to be a novel imaging technique for detecting new CNV or to follow CNV activity independent of CNV size and leakage. Recently, Takeda and colleagues have shown early detection of experimental CNV, not visible on FA, using a similar technique with anti-CCR3 antibody fragments [41]. The advantage of using NPs over immunoconjugate dyes in bioimaging and targeted therapy is that they are less likely to incite an immune reaction.

In the treatment arm of the study, we were able to demonstrate significant reduction of CNV size and leakage by targeted gene therapy using NP coupled to ATPμ-Raf, a dominant negative form of Raf kinase (Figure 3, 4). Previously Singh *et al* treated experimental CNV using poly-lactide-co-glycolide (PLGA) nanoparticles carrying a VEGF inhibitory gene [42]. Our approach is different in a number of ways including the type of particles used, specificity of targeting, and the gene delivered. The particles that Singh *et al* used had a negative z-potential as expected for PLGA nanoparticles, our therapy has a neutral charge and therefore is not toxic in tissue-cultured cells. Our nanoparticles are smaller (45.3 nm vs. 270–420 nm) and form a stable shell through covalent bonds that are far more stable in blood circulation than polymer based (used by Sing *et al* and hydrolyzed in aqueous environment) or liposome based delivery systems. This stability may lead to more efficacy and/or more side effects. A separate toxicity study is planned to answer this question. The targeted gene, Raf kinase, is an integral member of an intracellular signal transduction pathway involved in regulation of the cell cycle. ATPμ-Raf is a mutant form of Raf-l that fails to bind ATP and blocks the endothelial cell Raf activity *in vitro* [12], [29]. Raf-1 mutation has been linked to vascular defect and apoptosis during embryogenesis and gene therapy with ATPμ-Raf causes endothelial cell apoptosis and tumor regression in rats [12], [43]. Our results indicate a similar mechanism of CNV regression, through induction of apoptosis in neovascular endothelial cells, after targeted gene therapy with NP-ATPμ-Raf. There were significantly higher number of TUNEL positive cells in the treated CNV as compared to the controls (Figure 5). Moreover, while the recruitment of macrophages per lesion decreased with time in both groups, more macrophage infiltration was noted on days 5 and 7 in the treatment group (Figure 6). The initial spike in macrophage infiltration is likely the result of the inflammatory response to the laser injury. The increased macrophage recruitment to the treated CNV closely follows the increased apoptotic activity, suggesting that the macrophages may be responding to cell death by apoptosis or necroptosis.

Repeated systemic administration of NP for the treatment of CNV may lead to side effects such as blood clots in the elderly patient population. However, Intravenous (I.V.) administration of our treatment is not repetitious as apposed to the intravitreal injections of anti-VEGF therapies for example. In addition, for some patients, I.V. injections are less invasive than intravitreal injections and do not carry the potential ocular complications. Furthermore, due to the specificity of the binding site, the dose of nanoparticles needed is small and the tissue distribution limited, making systemic side effects less likely. In our study, we did not attempt the intreavitreal route of delivery as part of the investigation. Due to the large size of the NP (45.3 nm), it is unclear if it can distribute through the vitreous and cross the retina to reach the lumen of the CNV vessels through phagocytosis, pinocytosis or other mechanisms.

We also explored the timing of imaging and treatment with targeted NP in our study. Although we were able to target CNV with NP and capture GFP expression by NP-angiography up to three weeks after CNV induction, the maximum efficacy of treatment was achieved when treatment was given on days 1,3 and 5 (42% reduction in CNV size). We saw a modest decrease in the CNV size (24.6%) when the treatment was given on days 3, 5 and 7 and found no effect with treatment on days 7, 9 and 11. This data is consistent with the timing of integrin αvβ3 expression in the laser induced CNV in rats [44]. Integrin αvβ3 is expressed up to 4 weeks after laser injury, however its expression levels are significantly higher in the early stages of CNV formation, peaking at day 7 [44]. The fact that we were unable to show CNV regression with treatment starting at later time points may be due to the concentrations of NP used or the efficacy of transfecting the CNV endothelial cells with ATPμ-Raf. Integrin αvβ3 expression has been shown in pathologic specimens from human CNV and diabetic retinopathy [23], [45], however, the timing and duration of expression of this integrin has not been studied.

In summary, our results provide evidence that systemic administration of αvβ3 targeted NP can be used to label the abnormal blood vessels of CNV for imaging and targeted gene therapy with ATPμ-Raf. These results provide a proof-of-concept for this emerging technology and encourage further experimentation to discern the integration of NPs into current imaging and treatment of CNV and retinal neovascularization. Large animal studies and experiments utilizing NP coupled to various dyes and chemotherapeutic or anti-neovascular compounds need to be pursued to further explore the efficacy of this diagnostic and treatment strategy.

## References

[1] Pascolini D, Mariotti SP, Pokharel GP, Pararajasegaram R, Etya'ale D (2004). 2002 global update of available data on visual impairment: a
compilation of population-based prevalence studies.. Ophthalmic Epidemiol.

[2] Congdon N, O'Colmain B, Klaver CC, Klein R, Muñoz B (2004). Causes and prevalence of visual impairment among adults in the
United States.. Arch Ophthalmol.

[3] Jager RD, Mieler WF, Miller JW (2008). Age-related macular degeneration.. N Engl J Med.

[4] Ferris FL, Fine SL, Hyman L (1984). Age-related macular degeneration and blindness due to neovascular
maculopathy.. Arch Ophthalmol.

[5] Seddon JM, Chen CA (2004). The epidemiology of age-related macular
degeneration.. Int Ophthalmol Clin.

[6] Blinder KJ, Bradley S, Bressler NM, Bressler SB, Donati G (2003). Effect of lesion size, visual acuity, and lesion composition on
visual acuity change with and without verteporfin therapy for choroidal
neovascularization secondary to age-related macular degeneration: TAP and
VIP report no. 1.. Am J Ophthalmol.

[7] D'Amato RJ, Adamis AP (1995). Angiogenesis inhibition in age-related macular
degeneration.. Ophthalmology.

[8] Gragoudas ES, Adamis AP, Cunningham ET, Feinsod M, Guyer DR (2004). Pegaptanib for neovascular age-related macular
degeneration.. N Engl J Med.

[9] Rosenfeld PJ, Brown DM, Heier JS, Boyer DS, Kaiser PK (2006). Ranibizumab for neovascular age-related macular
degeneration.. N Engl J Med.

[10] Gaudreault J, Fei D, Rusit J, Suboc P, Shiu V (2005). Preclinical pharmacokinetics of Ranibizumab (rhuFabV2) after a
single intravitreal administration.. Invest Ophthalmol Vis Sci.

[11] Bressler NM (2009). Antiangiogenic approaches to age-related macular degeneration
today.. Ophthalmology.

[12] Hood JD, Bednarski M, Frausto R, Guccione S, Reisfeld RA (2002). Tumor regression by targeted gene delivery to the
neovasculature.. Science.

[13] Guccione S, Li KC, Bednarski MD (2004). Molecular imaging and therapy directed at the neovasculature in
pathologies. How imaging can be incorporated into vascular-targeted delivery
systems to generate active therapeutic agents.. IEEE Eng Med Biol Mag.

[14] Kobayashi H, Lin PC (2006). Nanotechnology for antiangiogenic cancer therapy.. Nanomedicine (Lond).

[15] Thomson H, Lotery A (2009). The promise of nanomedicine for ocular disease.. Nanomedicine (Lond).

[16] Yancopoulos GD, Klagsbrun M, Folkman J (1998). Vasculogenesis, angiogenesis, and growth factors: ephrins enter
the fray at the border.. Cell.

[17] Brooks PC, Montgomery AM, Rosenfeld M, Reisfeld RA, Hu T (1994). Integrin alpha v beta 3 antagonists promote tumor regression by
inducing apoptosis of angiogenic blood vessels.. Cell.

[18] Tucker GC (2003). Alpha v integrin inhibitors and cancer therapy.. Curr Opin Investig Drugs.

[19] Kumar CC, Armstrong L, Yin Z, Malkowski M, Maxwell E (2000). Targeting integrins alpha v beta 3 and alpha v beta 5 for
blocking tumor-induced angiogenesis.. Adv Exp Med Biol.

[20] Stromblad S, Cheresh DA (1996). Integrins, angiogenesis and vascular cell
survival.. Chem Biol.

[21] Scatena M, Giachelli C (2002). The alpha(v)beta3 integrin, NF-kappaB, osteoprotegerin
endothelial cell survival pathway. Potential role in
angiogenesis.. Trends Cardiovasc Med.

[22] Luna J, Tobe T, Mousa SA, Reilly TM, Campochiaro PA (1996). Antagonists of integrin alpha v beta 3 inhibit retinal
neovascularization in a murine model.. Lab Invest.

[23] Friedlander M, Theesfeld CL, Sugita M, Fruttiger M, Thomas MA (1996). Involvement of integrins alpha v beta 3 and alpha v beta 5 in
ocular neovascular diseases.. Proc Natl Acad Sci U S A.

[24] Hammes HP, Brownlee M, Jonczyk A, Sutter A, Preissner KT (1996). Subcutaneous injection of a cyclic peptide antagonist of
vitronectin receptor-type integrins inhibits retinal
neovascularization.. Nat Med.

[25] Kamizuru H, Kimura H, Yasukawa T, Tabata Y, Honda Y (2001). Monoclonal antibody-mediated drug targeting to choroidal
neovascularization in the rat.. Invest Ophthalmol Vis Sci.

[26] Honda S, Nagai T, Negi A (2009). Anti-angiogenic effects of non-peptide integrin alphavbeta3
specific antagonist on laser-induced choroidal neovascularization in
mice.. Graefes Arch Clin Exp Ophthalmol.

[27] Berinstein A, Roivainen M, Hovi T, Mason PW, Baxt B (1995). Antibodies to the vitronectin receptor (integrin alpha V beta 3)
inhibit binding and infection of foot-and-mouth disease virus to cultured
cells.. J Virol.

[28] Wickham TJ, Mathias P, Cheresh DA, Nemerow GR (1993). Integrins alpha v beta 3 and alpha v beta 5 promote adenovirus
internalization but not virus attachment.. Cell.

[29] Heidecker G, Huleihel M, Cleveland JL, Kolch W, Beck TW (1990). Mutational activation of c-raf-1 and definition of the minimal
transforming sequence.. Mol Cell Biol.

[30] Zacks DN, Ezra E, Terada Y, Michaud N, Connolly E (2002). Verteporfin photodynamic therapy in the rat model of choroidal
neovascularization: angiographic and histologic
characterization.. Invest Ophthalmol Vis Sci.

[31] She H, Nakazawa T, Matsubara A, Hisatomi T, Young TA (2007). Reduced photoreceptor damage after photodynamic therapy through
blockade of nitric oxide synthase in a model of choroidal
neovascularization.. Invest Ophthalmol Vis Sci.

[32] Renno RZ, Terada Y, Haddadin MJ, Michaud NA, Gragoudas ES (2004). Selective photodynamic therapy by targeted verteporfin delivery
to experimental choroidal neovascularization mediated by a homing peptide to
vascular endothelial growth factor receptor-2.. Arch Ophthalmol.

[33] Sakurai E, Taguchi H, Anand A, Ambati BK, Gragoudas ES (2003). Targeted disruption of the CD18 or ICAM-1 gene inhibits choroidal
neovascularization.. Invest Ophthalmol Vis Sci.

[34] Marneros AG, She H, Zambarakji H, Hashizume H, Connolly EJ (2007). Endogenous endostatin inhibits choroidal
neovascularization.. FASEB J.

[35] Zambarakji HJ, Nakazawa T, Connolly E, Lane AM, Mallemadugula S (2006). Dose-dependent effect of pitavastatin on VEGF and angiogenesis in
a mouse model of choroidal neovascularization.. Invest Ophthalmol Vis Sci.

[36] Nakazawa T, Matsubara A, Noda K, Hisatomi T, She H (2006). Characterization of cytokine responses to retinal detachment in
rats.. Mol Vis.

[37] Gaudana R, Ananthula HK, Parenky A, Mitra AK (2010 Sep). Ocular Drug Delivery.. AAPS J.

[38] Robbins SG, Brem RB, Wilson DJ, O'Rourke LM, Robertson JE (1994). Immunolocalization of integrins in proliferative retinal
membranes.. Invest Ophthalmol Vis Sci.

[39] Brooks PC, Strömblad S, Klemke R, Visscher D, Sarkar FH (1995). Antiintegrin alpha v beta 3 blocks human breast cancer growth and
angiogenesis in human skin.. J Clin Invest.

[40] Brooks PC, Clark RA, Cheresh DA (1994). Requirement of vascular integrin alpha v beta 3 for
angiogenesis.. Science.

[41] Takeda A, Baffi JZ, Kleinman ME, Cho WG, Nozaki M (2009). CCR3 is a target for age-related macular degeneration diagnosis
and therapy.. Nature.

[42] Singh SR, Grossniklaus HE, Kang SJ, Edelhauser HF, Ambati BK (2009). Intravenous transferrin, RGD peptide and dual-targeted
nanoparticles enhance anti-VEGF intraceptor gene delivery to laser-induced
CNV.. Gene Ther.

[43] Hüser M, Luckett J, Chiloeches A, Mercer K, Iwobi M (2001). MEK kinase activity is not necessary for Raf-1
function.. EMBO J.

[44] Tang R, Long J, Chen B (2009). Expression of integrin alphavbeta3, tissue factor, and vascular
endothelial growth factor in experimental choroidal
neovascularization.. Zhong Nan Da Xue Xue Bao Yi Xue Ban.

[45] Ning A, Cui J, Maberley D, Ma P, Matsubara J (2008). Expression of integrins in human proliferative diabetic
retinopathy membranes.. Can J Ophthalmol.

